# Decade of Twist Channel Angular Pressing: A Review

**DOI:** 10.3390/ma13071725

**Published:** 2020-04-07

**Authors:** Adéla Macháčková

**Affiliations:** Faculty of Materials Science and Technology, VŠB–Technical University of Ostrava, 17. Listopadu 15, 708 00 Ostrava, Czech Republic; adela.machackova@vsb.cz; Tel.: +420-597-324-344

**Keywords:** twist channel angular pressing, severe plastic deformation, microstructure, mechanical properties

## Abstract

The methods of severe plastic deformation (SPD) have gained attention within the last decades primarily owing to their ability to substantially refine the grains within metallic materials and, therefore, significantly enhance the properties. Among one of the most efficient SPD methods is the equal channel angular pressing (ECAP)-based twist channel angular pressing (TCAP) method, combining channel twist and channel bending within a single die. This unique die affects the processed material with three independent strain paths during a single pass, which supports the development of substructure and efficiently refines the grains. This review is intended to summarize the characteristics of the TCAP method and its main features documented within the last decade, since its development in 2010. The article is supplemented with a brief characterization of other known SPD methods based on the combination of ECAP and twist extrusion (TE) within a single die.

## 1. The Principles of Severe Plastic Deformation

Rapid development of novel construction components, innovative tools, and modern features in the industry and commerce goes hand in hand with the need to research materials with enhanced performance and increased longevity. Generally, the mechanical, physical, and utility properties of metallic materials are strongly affected by their structures—grain size in particular. By this reason, the ultra-fine grained (UFG) and nanomaterials featuring grain sizes between 100 nm and 1 µm and 1 nm and 100 nm, respectively, have been developed and intensively researched.

The Hall–Petch relation, although expressed in various ways according to the considered structural features and deformation history of the examined material [1], generally expresses the reciprocal proportion of the strength of the material and grain size (Equation (1)),

(1) $$\sigma =\sigma_{0}+k\cdot d^{-\frac{1}{2}}$$

where *σ*_0_ and *k* are constants depending on the material chemical composition and deformation history, and *d* is the grain size.

In other words, the smaller the grains, the higher the strength. Despite the fact that this relation only applies for grain sizes above the critical value, the ratio of grain boundaries to grain interiors below which increases rapidly (which leads to the grain boundary sliding phenomenon), the validity of the Hall–Petch relation has been proven even for metals with grain sizes between 10 and 100 nm [1]. Nevertheless, such a fine microstructure is hardly achievable by any known method of plastic deformation. Therefore, having in mind the Hall–Petch relation, methods of severe plastic deformation (SPD) have been developed.

The principle of SPD methods lies in modifying microstructures of bulk samples without significantly changing their external shapes. SPD methods can thus generally be considered to positively affect the strength of processed metallic materials via grain refinement. During SPD processing, free material flow is restricted as the stress state aimed to be achieved and maintained during processing is the hydrostatic pressure; such a stress state is necessary for a high density of defects to be introduced to the crystal lattice. This phenomenon is required to achieve a significant grain refinement without reducing the cohesion of the processed material. However, the resulting material’s performance not only depends on the achieved grain size, but also on substructure development, as it is a function of the density of dislocations and their (re)arrangement into specific shapes—networks.

The efficiency of SPD processes is ensured by two main parameters, which generally characterize them, as follows:

(1) Severe shear strain—regardless of the selected processing method, the shape of the die, and the number and character of the strain paths affecting the processed sample, all SPD methods are based on imposing severe shear strain into the material;

(2) Processing temperature—the temperature should be kept under the recrystallization temperature, that is, lower than 0.4–0.5 of the melting temperature of the particular metal, to aggravate recrystallization-induced grain growth and support grain refinement via accumulation of dislocations, formation of dislocation cells and walls, and subsequent polygonization, that is, formation of subgrains, which finally transform to fine new grains featuring high angle grain boundaries (HSGBs) [2]. Nevertheless, the substantial amount of energy introduced via the severe shear strain can impart recrystallization, resulting in grain fragmentation even at low temperatures [3]; the grain refining mechanisms that can occur in severely deformed metallic materials were thoroughly characterized by Glezer and Metlov [4]. However, the occurring grain refinement mechanisms also depend on the character of the processed metal (e.g., its stacking fault energy value), and the original material state, grain size, and deformation history in particular [5]. For example, metals with FCC lattices were documented to primarily deform via dislocation glide and twinning, but their ratio primarily depends on the applied strain rate and processing temperature; decreasing temperature and/or increasing strain rate support the dominance of twinning.

## 2. Severe Plastic Deformation Processes

The first ever attempt to perform severe plastic deformation via combinations of high pressure and severe shear strain was by professor P.W. Bridgman, who received the Nobel Prize in Physics in 1946 [6] (this idea later resulted in the invention of the high pressure torsion (HPT) process [7,8,9]). The breakthrough in SPD processing came later, in the 1980s, when Dr. V. M. Segal introduced the method of equal channel angular pressing (ECAP) [10,11,12] to the world.

In the early beginnings, a few processes were developed, some of which were also adjusted to enable processing of hollow billets (tubes), as well as powders and composite materials. However, the human ambition to improve the existing led to a massive development of SPD techniques. The everlasting research has brought about not only advancements of the already introduced methods, but also the design and subsequent verification of new methods, all of which have led to gradual amelioration of the original processes and tools and/or equipment geometry, allowing processing of not only typical bulk metallic materials, but also materials with low formability or hard-to-deform metals [13]. Although numerous SPD processes have been introduced, not all the existing methods can be listed here owing to the continuing research in this field. However, the examples are high pressure sliding [14], high pressure tube twisting [15], friction stir processing [16], equal channel angular drawing [17], ECAP–partial back pressure [18], accumulative back extrusion [19], accumulative roll bonding [20], accumulative spin bonding [21], constrained groove pressing [22], constrained studded pressing [23], parallel tubular channel angular pressing [24], tube channel pressing [25], elliptical cross-section spiral equal-channel extrusion [26], C-shape equal channel reciprocating extrusion [27], half channel angular extrusion [28], cyclic extrusion compression [29], cyclic closed die forging [30], cyclic expansion extrusion [31], cyclic extrusion compression angular pressing [32], (variable lead) axi-symmetric forward spiral extrusion [33,34], forward shear normal extrusion [35], rotary extrusion [36], torsion extrusion (TE) [37], twist extrusion (TE) [38], simple shear extrusion [39], continuous shearing [40], continuous confined strip shearing [41], continuous frictional angular extrusion [42], repetitive corrugation straightening [43], severe torsion straining [44], and so on.

Each of the above-mentioned SPD methods have pros and cons. Nevertheless, all of them aim to impart substantial structure refinement and the best possible structure homogeneity via imposing severe shear strain. Certain methods, such as HPT, are even able to introduce nano-scale features and provoke the formation of nanostructure. Nevertheless, the ECAP method still remains one of the most researched SPD technologies and keeps inspiring researchers worldwide.

It is quite complicated to characterize SPD methods according to strict criteria. Among the main criteria characterizing the SPD methods are primarily whether they are continuous or discontinuous, and the maximum size of the sample that can be processed by the method [45]—for example, HPT is only suitable for discontinuous processing of thin, coin-like samples, whereas ECAP–conform is suitable for processing of bulk rods and can also be applied industrially [46]. From the viewpoint of process efficiency, the maximum possible imposed strain is crucial. Imposing the maximum shear strain via a single pass while keeping the sample consistent and defect-free is favourable. However, not only the amount of the imposed strain, but also its homogeneity across the cross section of the sample is an important factor that consequently affects the homogeneity of properties within the processed material, as well as the possible development of residual stress.

Similar to virtually all the SPD methods, the maximum amount of the shear strain that can be introduced during a single pass while maintaining reasonable cross-sectional homogeneity and consistency of the processed material is limited for the basic and most researched method of angular pressing—ECAP. Despite the fact that the final material performance can be affected via changing processing parameters (friction, temperature, die geometry, and so on), the required modifications of particular processing steps necessarily bring about demands on the used equipment and reduce process effortlessness. The process of twist extrusion (TE) advantageously introduces high shear strain (which can be modified by changing the twist angle *γ*_max_), but, on the other hand, also features high strain inhomogeneity across the cross section. Under optimized conditions, that is, having reduced/eliminated their negative aspects, both the ECAP and TE methods are very convenient for possible application in the industry. Considering these factors, combining the ECAP and TE methods is a favourable way to take advantage of the positive aspects of both and create an advantageous processing methodology for the preparation of ultra-fine-grained (UFG) materials. The combination of both can either be performed by consequent processing of the billet (which would require equipment for both the methods to be in hand, as well as optimization of the process so that the processed material is not affected by the dwells and varying processing conditions), or by introducing a novel single process. This solution preferably offers a wide possibility of variations while keeping the production process quick and easy. In accordance with the aim to improve the efficiency of SPD processing, the method of twist channel angular pressing was developed.

## 3. Twist Channel Angular Pressing

**Figure 1:**
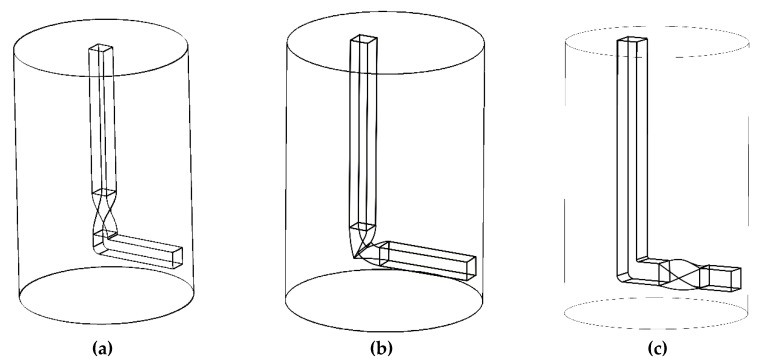
Experimentally investigated variants of twist channel angular pressing (TCAP): (**a**) Variant *I*, (**b**) Variant *II*, and (**c**) Variant *III*.

The method of twist channel angular pressing (TCAP) was firstly introduced by Kocich et al. in the 2010 [47]. TCAP is inspired by two individual SPD techniques, equal channel angular pressing (ECAP) and twist extrusion (TE), combining the positive aspects of both into a unique novel method. As mentioned above, ECAP and TE can be combined in several ways, as shown in Figure 1a to Figure 1c. Nevertheless, as experimentally proven [48], only one of the variants introduces reasonable processing parameters (e.g., punch load, temperature distribution) and preserves the constant shape of the sample, thus ensuring the repeatability of the entire process; Variant *I* (Figure 1a).

Although Variant *II* (Figure 1b) also combines both the ECAP and TE processes, this variant brings about several issues, probably the most severe of which is that positioning the twist directly to the bending introduces substantial local reduction of the channel cross section. In other words, the process then locally involves forward extrusion, which necessarily changes the processed sample cross section and introduces changes in its dimensions, by which the repeatability of the process, the crucial factor of all SPD processes, is disabled. This phenomenon can only be compensated by the application of back pressure. The second significant issue for this variant is a substantial increase in the punch load, which becomes even more substantial by the effect of applied back pressure. Among others, numerous cross-sectional reductions typical for this variant introduce a complex stress state, which can be unfavourable for the processed material.

The number of issues is reduced for Variant *III* (Figure 1c), although they are not eliminated. The main issue is related to the location of the twist being positioned behind the bending, which leads to a partially twisted final shape of the processed sample. Besides, by locating the twist behind the bending, the issues of the TE method, that is, partial cross-sectional deformation of the processed sample, as well as cross-sectional inhomogeneity of the imposed strain, are not eliminated; unlike Variant *I*, Variant *III* does not feature any final deformation zone that could suppress/reduce the strain inhomogeneity imparted by the twist.

**Figure 2:**
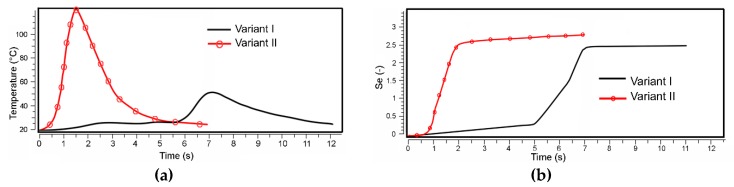
Comparison of deformation parameters: (**a**) temperature, (**b**) effective imposed strain.

Finite element analyses (FEAs) were further performed to evaluate the individual variants [45]. All of the modelled TCAP variants featured identical processing conditions, that is, room temperature processing with identical extrusion velocity, friction, and boundary conditions. The graphical dependencies depicted in Figure 2a,b show the differences between Variant *I* and Variant *II*. As shown by the temperature dependencies in Figure 2a, the increase in temperature for Variant *II* is more than twice as high as for Variant *I*, which can negatively affect the structures of processed materials featuring low melting temperatures. Moreover, the increase in temperature introduced by the development of deformation heat does not correspond to the increase in the imposed strain, which is only by approximately 20% (Figure 2b). By these reasons, only Variant *I* is further characterized (referred to just as TCAP).

**Figure 3:**
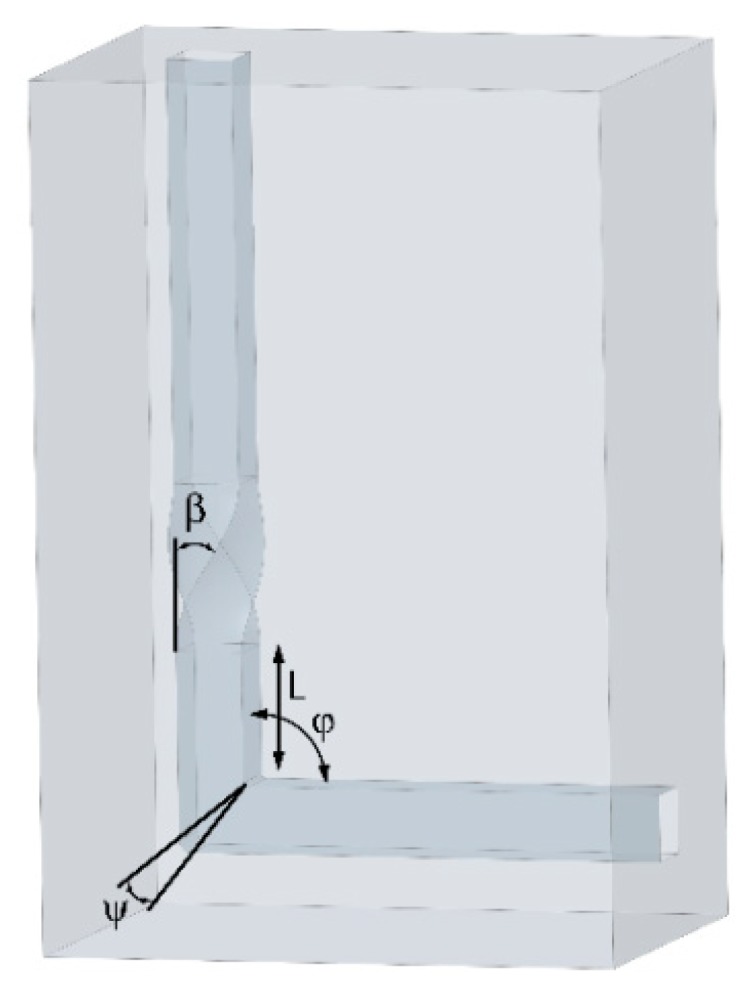
Schematic depiction of the TCAP die.

The TCAP die (Figure 3) consists of a single channel bent under the desired channel bending angle *φ*, preferably between 90° and 120° (depending on the processed material and its formability), and the first channel part contains a twist defined with multiple parameters, such as the twist slope angle *β*, twist rotation angle *ω*, and distance between the end of twist part within the channel and the channel bending *L*, all of which affect the final properties of the material and the amount and distribution of the imposed strain, as documented by the preliminary TCAP studies characterizing the method and describing its influence on the material behaviour, with the help of numerical modelling and subsequent practical experiments [47,49].

The most recent FEA study, which was applied to analyse mutual correlations of strain distribution and punch load, focused on the optimization of the TCAP die geometry from the viewpoint of minimizing the punch load while imposing a high shear strain [50]. Nine TCAP dies with varied twist slope angle *β* of 35°, 45°, and 55°, and channel bending angle *ψ* of 90°, 100°, and 110°, were designed. The results showed that, among all the combinations, the die with the twist slope angle of 45° and channel bending angle of 110° features a reasonable strain rate and relatively low punch load. The results were experimentally validated with sufficient correlation by performing TCAP of AA6061-T6 alloy. Moreover, to supplement the study with data on the effects of the processing temperature, the samples were processed at room temperature, 150 °C, and 250 °C; the sample processed at room temperature exhibited the best mechanical properties achieved.

### 3.1. Imposed Strain

The fact that the TCAP process consists of two individual methods has to be taken into account when calculating the mean value of the total imposed strain. In other words, the strain imposed in both the deformation zones, the twist and bending channel parts, has to be calculated separately.

#### 3.1.1. Channel Bending Part

**Figure 4:**
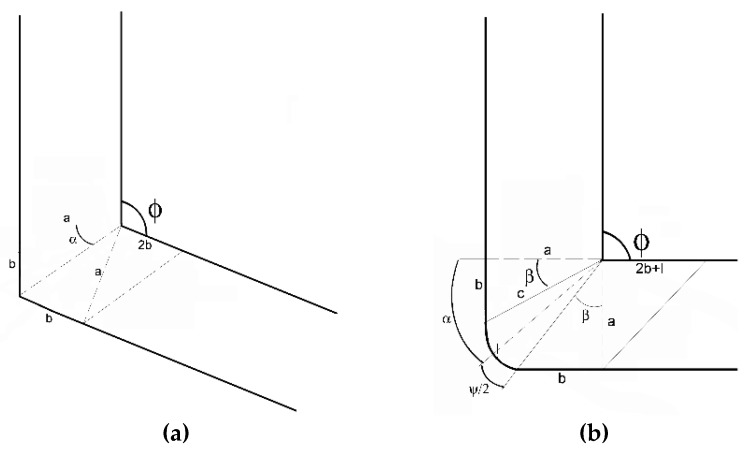

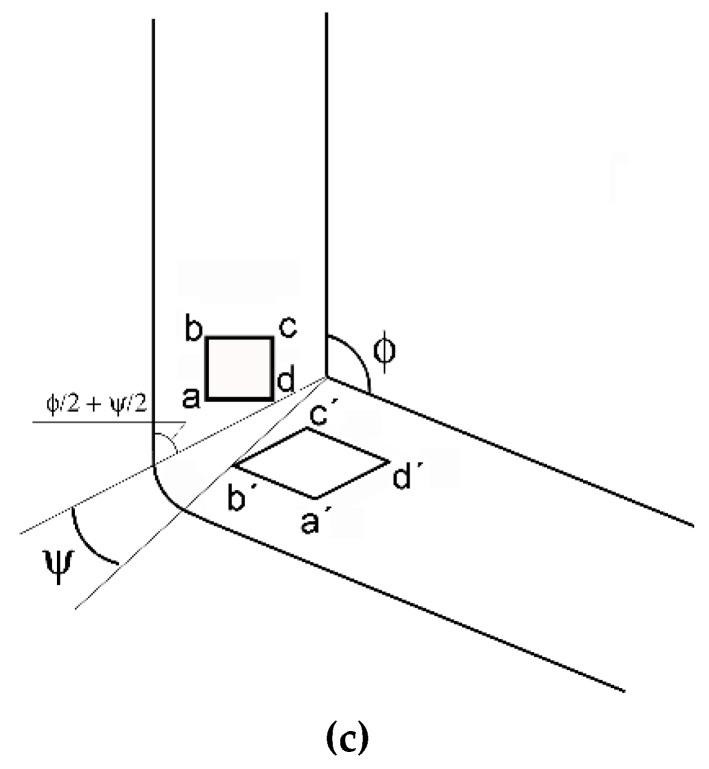
Schematic depiction of individual geometrical features for the following cases: (**a**) *ψ* = 0, (**b**) *ψ* ≠ 0; (**c**) effect of varying angle *ψ* on the individual deformation zone geometrical parameters.

The used symbols and the particular geometrical features are summarized in Figure 4a–c.

The following equations (Equations (2)–(4)) lead to the mathematical expression of the imposed shear strain shown in Equation (5) for the case when *ψ* = 0, that is, the outer channel bending radius is neglected. The used symbols are the die geometrical features, as referred to in Figure 4a.

(2) $$\alpha =\frac{\pi -\varphi }{2}=\frac{\pi }{2}-\frac{\varphi }{2}$$

(3) $$\frac{b}{a}=tg\alpha =\cot g\frac{\varphi }{2}$$

(4) $$\frac{b}{a}=\cot g\frac{\varphi }{2}\Rightarrow b=a\cdot \cot g\frac{\varphi }{2}$$

(5) $$\gamma =\frac{2b}{a}=\frac{2\cdot a\cdot \cot g\left(\frac{\varphi }{2}\right)}{a}=2\cdot \cot g\left(\frac{\varphi }{2}\right)$$

Considering the imposed strain to be $\varepsilon =\frac{\gamma }{\sqrt{3}}$, the final simplified equation for the calculation of the imposed shear strain is then expressed as Equation (6).

(6) $$\varepsilon =\frac{2}{\sqrt{3}}\cdot \cot g\left(\frac{\varphi }{2}\right)$$

For the case of *ψ* ≠ 0, that is, when the outer channel bending radius is not neglected, the following equations, Equations (7)–(10), ensue from Equation (2), and from angle *β*, which is expressed as follows:

(7) $$\beta =\alpha -\frac{\psi }{2}=\frac{\pi }{2}-\frac{\varphi }{2}-\frac{\psi }{2}$$

(8) $$\frac{b}{a}=tg\beta =\cot g\left(\frac{\varphi }{2}+\frac{\psi }{2}\right)$$

(9) $$\frac{a}{c}=\cos\beta =\cos\left(\frac{\pi }{2}-\frac{\varphi }{2}-\frac{\psi }{2}\right)=\sin\left(\frac{\varphi }{2}+\frac{\psi }{2}\right)\Rightarrow c=\frac{a}{\sin\left(\frac{\varphi }{2}+\frac{\psi }{2}\right)}=a\cdot \cos ec\left(\frac{\varphi }{2}+\frac{\psi }{2}\right)$$

(10) $$l=\psi \cdot c=\psi \cdot a\cdot \cos ec\left(\frac{\varphi }{2}+\frac{\psi }{2}\right)$$

The final equation for the calculation of the imposed shear strain is then expressed as Equation (11), resp. Equation (12).

(11) $$\gamma =\frac{2b+l}{a}=\frac{2b}{a}+\frac{l}{a}=2\cdot \cot g\left(\frac{\varphi }{2}+\frac{\psi }{2}\right)+\frac{\psi \cdot a\cdot \cos ec\left(\frac{\varphi }{2}+\frac{\psi }{2}\right)}{a}$$

(12) $$\gamma =2\cdot \cot g\left(\frac{\varphi }{2}+\frac{\psi }{2}\right)+\psi \cdot \cos ec\left(\frac{\varphi }{2}+\frac{\psi }{2}\right)$$

Considering the imposed strain to be $\varepsilon =\frac{\gamma }{\sqrt{3}}$, the final simplified equation for the calculation of the imposed shear strain is then expressed as Equation (13).

(13) $$\varepsilon =\frac{1}{\sqrt{3}}\cdot \left[2\cdot \cot g\left(\frac{\varphi }{2}+\frac{\psi }{2}\right)+\psi \cdot \cos ec\left(\frac{\varphi }{2}+\frac{\psi }{2}\right)\right]$$

All of the above mentioned equations calculate the assumption of simplified boundary conditions and constant processing parameters, such as homogeneous plastic flow, ideally plastic material behaviour, absence of friction between the sample and die, complete filling of the channel with extruded material, and so on. In other words, certain parameters having complex effects, such as widening of the deformation zone, are usually neglected. Implementing the Segal theory of slip lines [51] to predict widening of the deformation zone, the angle characterizing the boundaries of the deformation zone can be depicted as *β* for dies with no outer channel bending radius (*Ψ* = 0); angle *β* = 0 when neglecting friction and increases up to *β* = *π*/2 with increasing friction. Assuming that the material exhibits ideal plastic flow, the deformation zone boundaries are given by angle *α*, that is, *β* = *π*/2 – 2*α* for angle *Φ* = 90°. On the basis of these facts, the imposed strain for dies featuring angle *Φ* = 90° can be expressed via Equation (14):

(14) $$\varepsilon =\sqrt{\frac{1}{3}}\sqrt{1+2a(1-b+a)+b^{2}}$$

where *a* = 2*α*, *b* = (1 + *β*)*tgα*.

The *ε* value increases from 0.5774 for *β* = *π*/2, up to 1.1547 for *β* = 0. Simple shear occurring in the plane of intersection of both channel parts (*β* = 0), as well as shear occurring behind the deformation zone for dies featuring the outer channel bending radius (*Ψ* > 0), should, theoretically, impart homogenous shear strain distribution [52]. Nevertheless, deformation hardening occurring during ECAP processing leads to asymmetrical distribution of the strain rate in the deformation zone region, which results in the development of a dead zone and occurrence of strain heterogeneity. However, friction can affect the size of the dead zone; increasing friction between the die and sample surfaces attributes to decreasing the size of the dead zone region. In case that the die is filled with the processed material completely, the shape and dimensions of the deformation zone region are primarily given by the die geometry, that is, *Ψ* angle; friction has a neglectable effect. Assuming elimination of the dead zone, friction affects the imposed strain locally at the surface of the processed sample, which increases strain heterogeneity across the sample cross section. On the contrary, if a dead zone is present, friction affects the imposed strain throughout the entire sample volume. Increasing friction generally increases the imposed strain heterogeneity, decreases angle *β*, and decreases size of the dead zone region. Increasing the strain hardening rate results in increasing angle *Ψ* and increasing angle *Ψ*, and increasing the size of the dead zone region increases angle *β*. Generally, however, *Ψ* ≠ *β.*

#### 3.1.2. Channel Twist Part

**Figure 5:**
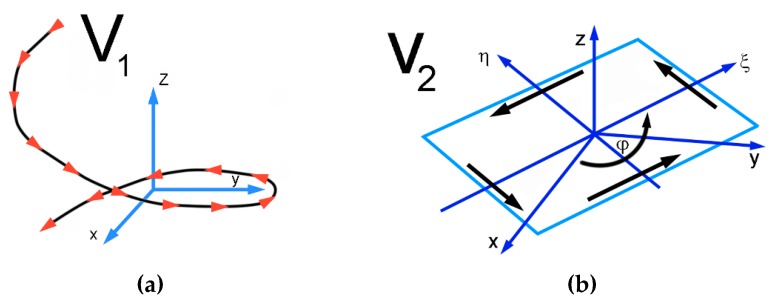
Schematic depiction of individual material flow velocities: (**a**) *v*_1_, (**b**) *v*_2_.

For the channel part featuring the twist, calculation of the imposed strain has to be performed considering, among others, different flow velocities in the particular material regions. The individual *v_1_* and *v_2_* components of plastic flow velocity in the twist channel part are schematically depicted in Figure 5a,b, respectively, and mathematically expressed via the following equations.

The individual plastic flow velocity components in the individual directions can be expressed as follows (Equations (15)–(20)) [53]:

(15) $$v_{1x}=-\frac{yv_{0}tg\gamma }{R}$$

(16) $$v_{1y}=\frac{xv_{0}tg\gamma }{R}$$

(17) $$v_{1z}=v_{0}$$

(18) $$v_{2x}=\frac{\partial \left(\Omega P\right)}{\partial y}$$

(19) $$v_{2y}=-\frac{\partial \left(\Omega P\right)}{\partial x}$$

(20) $$v_{2z}\equiv 0$$

where Ω is a function defining the cross-sectional shape, Ω = 0 at the periphery of the sample cross section, Ω > 0 within the sample cross section, and Ω < 0 outside the cross section. *P* is a parameter characterizing the variable part of the velocity field; P is defined by variation methods and its value at the periphery of the sample cross section is /*P*/ = /*v*_2_/. *R* is half of the length of the sample’s edge.

The final velocity of material flow when passing through the twist channel part is then expressed via Equation (21):

*v* = *v*_1_ + *v*_2_ (21)

where *v_1_* is the velocity field component expressing flow of the cross section as a whole, whereas *v_2_* is the velocity field component expressing transversal material flow across the cross section.

The shear strain is subsequently expressed in dependence on the particular cross-sectional location within the processed material, regarding axis—cross-section periphery of the sample. The maximum component of the shear strain expressed via the *v_1_* velocity component, that is, neglecting the *v_2_* velocity component, is formulated as Equation (22):

(22) $$\gamma_{\max}=\sqrt{3}\cdot tg\beta_{\max}$$

where $\gamma_{\max}$ is the shear strain and $\beta_{\max}$ is the twist slope angle.

Consequently, the shear strain can in this case be expressed as Equation (23).

(23) $$\varepsilon_{\max}=tg\beta_{\max}$$

When the *v_2_* velocity component is not neglected, the shear strain is expressed as Equation (24):

(24) $$\gamma_{\min}=\sqrt{3}\cdot \left(0.4+0.1\cdot tg\beta_{\max}\right)$$

where $\gamma_{\min}$ is the shear strain and $\beta_{\max}$ is the twist slope angle.

The imposed shear strain can then be expressed as Equation (25).

(25) $$\varepsilon_{\min}=0.4+0.1\cdot tg\beta_{\max}$$

For the twist part of the TCAP channel, the value of the imposed shear strain can thus be calculated using both the Equations (23) and (25). However, the mentioned equations are only applicable for the axial and peripheral cross-sectional regions of the processed sample, respectively. Relations characterizing the shear strain imposed during TCAP processing in the near-axial and near-peripheral cross-sectional regions of the processed sample would involve combinations of both Equations (23) and (25), as the final equation would take into account increments from both locations, depending on the particular monitored region. The simplified assumption of simply adding up the individual increments from both locations can, however, only be made if no vortex-like material flow is presupposed. Nevertheless, the results of numerical predictions showed that the vortex-like flow is most probably the key factor contributing to final homogenization of the imposed strain across the sample cross section. The imposed strain is not homogeneous throughout the sample cross section after ECAP, especially for dies with corner radii, or dead zone regions. The smallest values of the imposed shear strain are recorded at the peripheral regions of the processed sample, that is, in regions adjacent to the outer channel radius. Similar phenomena, that is, the occurrence of a dead zone and shift of the deformation zone from the central die region, are also imparted via deformation hardening during ECAP, as proven by Kim et al. [52], who documented plastic flow velocity gradients to be present in both mentioned regions.

The mean value of the imposed shear strain is finally derived from relations for strain rates in the individual directions (Equations (26)–(30)):

(26) $${\dot{e}}_{xx}=P\frac{\partial^{2}\omega }{\partial x\partial y}$$

(27) $${\dot{e}}_{yy}=-P\frac{\partial^{2}\omega }{\partial x\partial y}$$

(28) $${\dot{e}}_{xy}=\frac{1}{2}P\left(\frac{\partial^{2}\omega }{\partial y^{2}}-\frac{\partial^{2}\omega }{\partial x^{2}}\right)$$

(29) $${\dot{e}}_{xz}=\frac{1}{2}\frac{\partial^{2}\left(\omega P\right)}{\partial y\partial z}-\frac{yV_{0}}{2R\cos^{2}\beta }\frac{d\beta }{dz}$$

(30) $${\dot{e}}_{yz}=-\frac{1}{2}\frac{\partial^{2}\left(\omega P\right)}{\partial x\partial z}+\frac{xV_{0}}{2R\cos^{2}\beta }\frac{d\beta }{dz}$$

Strain rate intensity from the above equations can further be expressed (Equation (31)).

(31) $$S\dot{e}=\frac{\sqrt{2}}{3}\sqrt{{\left({\dot{e}}_{xx}-{\dot{e}}_{yy}\right)}^{2}+{\left({\dot{e}}_{xx}-{\dot{e}}_{zz}\right)}^{2}+{\left({\dot{e}}_{zz}-{\dot{e}}_{yy}\right)}^{2}+6{\left({\dot{e}}_{xy}{}^{2}+{\dot{e}}_{xz}{}^{2}+{\dot{e}}_{yz}{}^{2}\right)}^{}}$$

Integration of Equation (31) consequently results in the mean value of the imposed strain for the twist part of the channel (Equation (32)):

(32) $$\varepsilon =\int S\dot{e}\;dt$$

The final relation defining the mean value of the imposed strain during TCAP process can eventually be expressed via Equation (33).

(33) $$\varepsilon =\frac{1}{\sqrt{3}}\cdot \left[2\cdot \cot g\left(\frac{\varphi }{2}+\frac{\psi }{2}\right)+\psi \cdot \cos ec\left(\frac{\varphi }{2}+\frac{\psi }{2}\right)\right]+\int S\dot{e}\;dt$$

### 3.2. Die Geometry

On the basis of not only the mentioned studies, TCAP enables to impose a substantially high effective strain into the material during a single pass (up to ~2.3), which significantly reduces the number of passes necessary to acquire a material with an ultra-fine (nano) structure, and thus makes the entire processing less time-consuming and more efficient [47]. As reported by Bagherpour et al. [5], the favourable combination of both the ECAP and TE methods introduces relatively large plastic strains into the processed material by combining different deformation modes, which eventually leads to very fine grains and a high fraction of high angle grain boundaries (HAGBs). In addition, the advantageous combination leads to homogenization of final properties throughout the sample cross section and length, while keeping the shape unchanged [47]. Sole application of TE leads to a certain permanent deformation of the processed sample (primarily imparted by the friction during processing). This phenomenon can be compensated by the application of back pressure, however, back pressure is not necessary to be applied during ECAP, that is, the use of back pressure can be eliminated by selecting the favourable die design, that is, TCAP Variant *I*. However, the overall efficiency of Variant *I* varies depending on the die geometry (see Figure 3). The distance between the end of twist part of the channel and the channel bending *L* was shown to have a neglectable influence on the amount of the imposed strain, but to substantially affect the strain homogeneity; increasing the distance between the twist and bending channel parts suppresses the vortex-like flow and improves strain homogeneity across the sample cross section. Regarding twist slope angle *β* and twist rotation angle *ω*, both parameters significantly affect both the amount and homogeneity of the imposed strain; higher values of *β* and *ω* angles result in higher imposed strain, but also in higher inhomogeneity. The channel bending angle *φ* especially affects the amount of the imposed strain. However, its negative effect on strain homogeneity via the formation of dead zones for large bending angles should be mentioned, too. In summary, the most significant effect on increasing the amount of the imposed strain was increasing the twist rotation angle *ω*, and mutually increasing the twist slope angle *β* and decreasing the channel bending angle *φ*. The fact that the dead zone corner gap is substantially smaller for the TCAP die than for ECAP die with identical channel bending angles *φ* needs to be stressed, too.

### 3.3. Strain Path

The description of the strain path affecting the material when passing through the TCAP die ensues from the simple shear model developed by Latypov et al. [54]. According to the model, the material processed through the ECAP die is subjected to simple shear in a limited intersection plane located in the channel bending; this model can be applied to describe the bending part of TCAP. The twist part of the die is then considered to be comparable to twist extrusion (TE), during which the material is subjected to simple shear in two intersecting planes. The TE process was already characterized to be more efficient than ECAP from the viewpoint of the occurring grain refinement at identical imposed strains owing to the presence of two independent shear strain paths [55]. However, the TCAP process imposes shear strain into the processed material along three independent shear planes, which ensures its higher efficiency compared with ECAP featuring a single shear plane, as well as TE having two shear planes.

**Figure 6:**
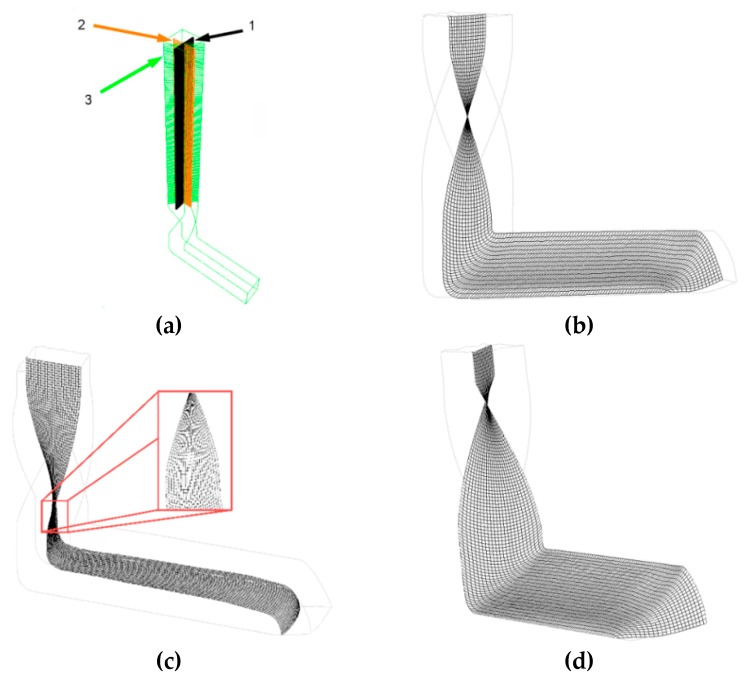
Predicted material flow through the TCAP die, three superimposed meshes: (**a**) detail of superimposed mesh *1* (**b**); superimposed mesh *2* (**c**); and superimposed mesh *3* (**d**).

Given by the three independent shear strain paths, the material flow during TCAP is rather complex [47]. Moreover, its intensity depends on the particular monitored location and selected cutting plane. Numerical prediction of the plastic flow during TCAP processing was performed using three superimposed meshes, as depicted in Figure 6a. As can be seen in the figure, all of the selected cutting planes with the superimposed meshes *1*, *2*, and *3* are situated to the sample extrusion axis and rotated by 45° from one another; this particular rotation is selected in accordance with the rotation imparted to the material by the twist channel part. Superimposed meshes *1* and *3*, depicted in Figure 6b,d, respectively, reveal the twist channel part to impart the most substantial plastic flow to the axial sample region (the plastic flow intensity decreases across the sample cross section towards its periphery). This flow inhomogeneity is then reduced in the channel bending part, the flow velocities of the axial and peripheral material regions in which equalize. Therefore, the imposed shear strain exhibits high homogeneity along the cutting planes depicted via superimposed meshes *1* and *3*. On the other hand, superimposed mesh *2*, the detail of which is depicted in Figure 6c, features differences when compared with meshes *1* and *3*. In this particular cutting plane, the plastic flow imparted by the twist channel part is more intense in the axial sample region than in its peripheral regions and, as a result of the vortex-like flow, this character of plastic flow remains visible even after passing through the channel bending. Among the significant factors affecting the plastic flow homogeneity is also the friction between the channel and extruded material; increasing friction not only increases the amount of the imposed shear strain, but also imparts flow inhomogeneities across the sample cross section [56].

**Figure 7:**
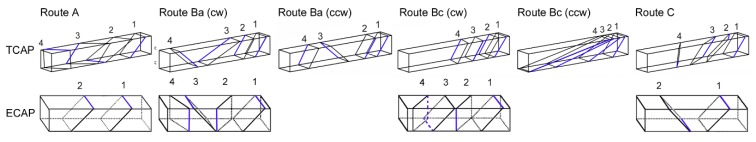
Comparison of activated shear systems during processing via multiple equal channel angular pressing (ECAP) and TCAP methods.

Similar to ECAP, TCAP can also be performed in multiple passes. However, given the unique strain path and complex material flow, the shear systems activated during ECAP and TCAP when applying the conventional ECAP routes are not identical. This was demonstrated by Kocich et al. [49], by performing individual experiments in which they applied the known ECAP deformation routes, that is, route *A* (no rotation between passes), route *Ba* (±90° rotation between passes), route *Bc* (+90° rotation between passes, clockwise, and counter-clockwise), and route *C* (180° rotation between passes), for both ECAP and TCAP. For example, processing of the sample via ECAP, route *A*, results in repetitive activation of identical shear systems. On the other hand, processing of the sample via TCAP, route *A*, activates multiple shear systems owing to the implemented twist channel part. Activation of different slip systems will also lead to proportional changes in the energy dissipation modes characteristic for the SPD processing, especially regarding the ratio of dynamic recrystallization and dislocation-disclination accommodation [57]. A comparison of the shear systems activated during processing via the ECAP and TCAP deformation routes in multiple passes is shown in Figure 7.

### 3.4. Structure and Properties

The general comparison of TCAP with the TE and ECAP technologies by which TCAP was inspired shows that TCAP ensures higher homogeneity as well as higher efficiency of the imposed strain across the cross section of the processed material.

The numerically predicted high homogeneity of the imposed strain throughout the sample when extruded with optimized processing parameters was validated by experimental investigations of mechanical properties. The stress–strain curves recorded during the tensile testing of specimens taken from the axial and peripheral regions of the commercial purity (CP) Cu TCAP-processed sample both reached the maximum value of 440 MPa, and their courses featured only minor deviations [47]. Similar trends were documented for the CP Cu microhardness, which increased by 80% after TCAP processing, to the average value of 108 HV, and exhibited only minor deviations along the measured diagonals across the square TCAP sample cross section [56].

**Figure 8:**
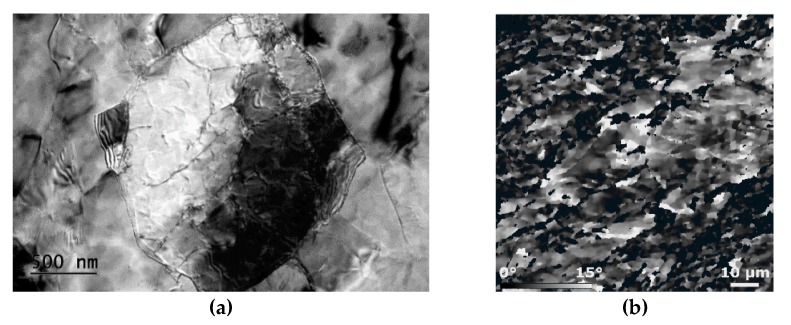
Transmission electron microscopy image showing the detail of CP Al substructure after single pass TCAP (**a**); residual stress depicted via internal grains misorientations in scale from 0° (black) to 15° (white) for the two-year-old CP Al sample (**b**).

The high efficiency of TCAP can primarily be attributed to the three independent shear strain paths, supporting development of lattice distortions acting as obstacles for dislocations movement, and nucleation sites for recovery. The comparison of stress–strain curves for CP Al processed via ECAP and TCAP showed the strength to significantly increase after a single ECAP pass, and then increase slightly again after the second pass via the *A* and *Bc* routes up to 300 MPa for the *Bc* route processed sample. Nevertheless, the sample processed via a single TCAP pass exhibited the strength of approximately 340 MPa, which can be attributed to two main phenomena—the presence of strengthening secondary particles, and substantial grain refinement. The efficiency of multiple TCAP on grain refinement was thoroughly documented for CP Cu [49], whereas the effects of the strain paths acting during a single pass through the TCAP die were characterized via studies on samples of CP Al [58,59]. The analyses showed that the original unprocessed Al sample exhibited relatively coarse grains with bimodal grain size distribution (the average grain diameter was 39 μm) and more or less random grains orientations. After a single ECAP pass, the grains refined significantly and featured the average diameter of slightly more than 10 μm. Processing by two ECAP passes via route *A* led to grain refinement to the average diameter of ~8 μm, while processing via two ECAP passes via route *Bc*, which is considered to be the most efficient ECAP route from the viewpoint of grain refinement [60], resulted in the average grain size of 7.4 μm. The most intensive grain refinement was recorded after TCAP, the average grain size after a single pass of which was ~5.8 µm (68% of grains were smaller than 5 µm). The grains also featured a high dislocations density and well developed substructure, as documented by Figure 8a [61]. The sample processed by two ECAP passes via route *Bc* also featured refined grains and a deformed substructure, but exhibited no highly developed subgrains [58].

Taking into consideration the high imposed energy and substructure development occurring during a single pass, the structure of TCAP-processed material can be considered to be metastable, and further structure changes can be supposed to occur over time. This presupposition was confirmed in a study dealing with two-year-old CP Al samples [62]. After natural ageing, the restoration processes provoked the grains to further refine down to the average grain size of ~1 μm. Natural ageing also supported precipitation of strengthening particles; small angle neutron scattering (SANS) analyses showed the particles to preferentially precipitate in orientations corresponding to the active shear strain paths. The aged material also exhibited relaxation of residual stress; the study depicts the distribution of residual stress (via internal grains misorientations in scale from 0° to 15°) within the structure of the CP Al TCAP sample right after processing, and after the two years of natural ageing. As evident in Fig. 8b, the misorientations from 0° to 15° in which are depicted in the black to white scale, the aged structure has a tendency to relax and homogenize the level of residual stress throughout the structure. Stress-free locations start to form within the aged sample, too. Evaluation of mechanical properties performed via mapping of microhardness throughout the naturally aged the CP Al sample revealed that the homogeneity of mechanical properties remains stable over time. The map showed a decrease in the average HV value resulting from the occurrence of restoration processes and residual stress relaxation, although it exhibited high homogeneity.

### 3.5. Texture

**Figure 9:**
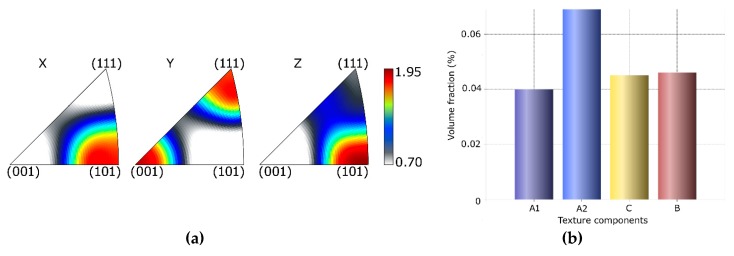
Inverse pole figures (**a**) and ideal texture components (**b**) for CP Al after single pass TCAP.

The comparison of textures within CP Al after TCAP and ECAP was also performed [58,59]; however, comparing textures of materials processed by different SPD processes can be ambiguous, as texture is affected by various factors and, above all, the strain paths along which the shear strain is imposed (ideal texture components are characterized according to the description performed by Beyerlein and Tóth [63]). The original weak cubic texture present in the unprocessed Al transformed into *A* ideal texture component, and *B* texture fibre parallel to the shear direction (<110>||SD) after a single pass ECAP; similar texture formation tendencies were also observed after two ECAP *Bc* route passes, whereas strong *A* ideal texture component {111} <011> formed in the structure after two ECAP *A* route passes. Processing via TCAP led to the formation of ideal <110>||SD and <100>||SD *B* texture fibres, as well as the *A* ideal texture component and *C* ideal texture component. Nevertheless, given the substantial substructure development and grain fragmentation, the maximum texture intensities were lower than two times random for all of the components, as also confirmed by a 3D EBSD (electron back-scattered diffraction) study [59]. Figure 9a shows inverse pole figures (IPFs) for the CP Al sample processed via a single pass TCAP, while Figure 9b depicts the intensities of the characteristic ideal texture orientations for this sample.

## 4. Twist Channel Multi Angular Pressing

The twist channel multi angular pressing (TCMAP) method was invented in order to explore the efficiency limits of TCAP. The TCMAP die basically consists of a TCAP die modified by implementation of another deformation section, that is, it contains multiple bendings. Kocich et al. [64] studied two different variants of the TCMAP die; the first variant consisted of the TCAP die supplemented with an additional bending situated between the original TCAP twist and bending channel parts, whereas the second variant consisted of the TCAP die having the additional channel bending located in front of the conventional TCAP twist and bending parts. 

The results of numerical simulations showed the imposed effective strain to increase when compared with the conventional TCAP. The maximum effective strain was higher for the second TCMAP variant having the additional bending implemented as the first die feature (maximum strain of ~3.2 compared with ~2.8 for the first variant having the additional bending between the TCAP twist and bending channel parts). However, the strain homogeneity was higher for the first variant. Among the positive aspects of TCMAP is the elimination of dead corner zones. On the other hand, the full contact of the extruded material with the channel, together with its complicated construction, increases the load on the punch, and consequently the power demands of the entire extrusion process. Measurements of microhardness values for the TCMAP-processed CP Al sample showed a substantial increase in the average HV value (increase by 97% compared with the original CP Al, to ~90 HV), as well as high microhardness homogeneity along the diagonals across the square TCMAP sample cross section [65].

## 5. Twisted Multi Channel Angular Pressing

The twisted multi channel angular pressing (TMCAP) method based on the combination of ECAP and TE was very recently presented by S. M. Alavizadeh et al. [66]. The TMCAP die consists of three independent parts, the first one of which is a rectangular extrusion channel with the length of 55 mm. The second part, bending under the angle of 156°, is followed by the third part being a twist with the rotation angle of 60° and total length of 16 mm. The last deformation feature within the extrusion channel is two bendings of 132° and 113°. The die is designed to enable the repeatability of the entire process.

Numerical predictions showed that the process imposes a substantially high effective strain onto the processed material, although the inhomogeneity of the imposed strain throughout the sample cross section is notable; the average effective imposed strain was predicted to be approximately 2.4, but the local strain maximum reached the value of 5. The inhomogeneity was also documented by the experimental microhardness testing performed on extruded CP Al. Nevertheless, selecting an optimized strain path while performing multiple TMCAP contributed to homogenization of the imposed strain and, correspondingly, the mechanical properties throughout the sample cross section. The yield strength of the processed AL1050 alloy increased by 164 % after four TMCAP passes.

## 6. Planar Twist Channel Angular Extrusion

Last, but not least, the planar twist channel angular extrusion (PTCAE) method is mentioned [67]. This process combines the ECAP technique with the planar twist extrusion (PTE) method within a single die, and thus simultaneously imposes severe shear strain to the processed material in three perpendicular planes within a single deformation zone. In the deformation zone, the processed sample is subjected to severe deformation from its original square cross-sectional shape at the channel inlet, to the parallelogram shape with the maximum distortion angle α in middle part of the channel, and finally returns to the square shape at the channel outlet [68]. Further, the processed material is subjected to shear strain, similar to ECAP, in the plane parallel to the two intersected channels. The die is designed to enable processing of the sample via multiple passes.

As proven by experiments with CP Al, the PTCAE process features two main advantages over the ECAP process; not only is it more efficient, but it also features high homogeneity of the imposed shear strain, and consequent uniformity of the mechanical properties; microhardness increased from the original 29 HV to 41 HV after processing via ECAP, and to 49 HV after one pass of PTCAE [69]. The efficiency of the entire process can primarily be optimized via changing the planar twist angle α, as shown by numerical simulations; decreasing the planar twist angle decreases the imposed effective strain and increases the cross-sectional area of the deformed sample. Further, increasing the planar twist angle results in increasing the deformation load owing to the higher strain imposed along the planar twist path in the deformation zone, and higher friction increased by the increased contact surface of the die and processed sample.

## 7. Conclusions

The review was focused on characterization of the main features of the equal channel angular pressing (ECAP)-based twist channel angular pressing (TCAP) method of severe plastic deformation (SPD). The unique construction of the die combining twist and bending in a single channel enables to impart severe shear strain into the processed material via three independent shear strain paths, which makes the process more efficient from the viewpoint of grain refinement and improvement of mechanical properties than ECAP. These conclusions were supported by numerous experimental studies, all documenting the efficiency of the TCAP process via substantial grain refinement and enhancement of mechanical properties. Numerical predictions of plastic flow via three independent superimposed meshes, together with characterization of the effects of the individual TCAP die features on the material behaviour, as well as calculations of the imposed shear strain, were performed as well. The study was supplemented with data characterizing the behaviour of CP Al samples processed via TCAP and subjected to two years of natural ageing, after which structure restoration, relaxation of residual stress, and grain refinement down to the ultra-fine scale that were observed. Having characterized the TCAP method, the twist channel multi angular pressing (TCMAP) and twisted multi channel angular pressing (TMCAP) methods featuring the combinations of TE and ECAP with the implementation of additional deformation features were presented. Last, but not least, the planar twist channel angular extrusion (PTCAE) method combining ECAP with planar twist extrusion (PTE) was presented.

## References

[1] Hansen N. (2004). Hall–Petch relation and boundary strengthening. Scr. Mater..

[2] Verlinden B., Driver J., Samajdar I., Doherty R.D. (2007). Thermo-Mechanical Processing of Metallic Materials.

[3] Fang Y., Chen X., Madigan B., Cao H., Konovalov S. (2016). Effects of strain rate on the hot deformation behavior and dynamic recrystallization in China low activation martensitic steel. Fusion Eng. Des..

[4] Glezer A.M., Metlov L.S. (2010). Physics of megaplastic (Severe) deformation in solids. Phys. Solid State.

[5] Bagherpour E., Pardis N., Reihanian M., Ebrahimi R. (2019). An overview on severe plastic deformation: Research status, techniques classification, microstructure evolution, and applications. Int. J. Adv. Manuf. Technol..

[6] Langdon T.G. (2013). Twenty-five years of ultrafine-grained materials: Achieving exceptional properties through grain refinement. Acta Mater..

[7] Qian C., He Z., Liang C., Cha Y., Ji W. (2020). Superior intracluster conductivity of metallic lithium-ion battery anode achieved by high-pressure torsion. Mater. Lett..

[8] Voronova L.M., Chashchukhina T.I., Gapontseva T.M., Krasnoperova Y.G., Degtyarev M.V., Pilyugin V.P. (2016). Effect of the deformation temperature on the structural refinement of BCC metals with a high stacking fault energy during high pressure torsion. Russ. Metall..

[9] Kunčická L., Lowe T.C., Davis C.F., Kocich R., Pohludka M. (2015). Synthesis of an Al/Al_2_O_3_ composite by severe plastic deformation. Mater. Sci. Eng. A.

[10] Kocich R., Greger M., Macháčková A. (2010). Finite element investigation of influence of selected factors on ECAP process. Proceedings of the METAL 2010: 19th International Metallurgical and Materials Conference, Roznov pod Radhostem.

[11] Elhefnawey M., Shuai G.L., Li Z., Nemat-Alla M., Zhang D.T., Li L. (2020). On achieving superior strength for Al–Mg–Zn alloy adopting cold ECAP. Vacuum.

[12] Hlaváč L.M., Kocich R., Gembalová L., Jonšta P., Hlaváčová I.M. (2016). AWJ cutting of copper processed by ECAP. Int. J. Adv. Manuf. Technol..

[13] Valiev R.Z., Langdon T.G. (2006). Principles of equal-channel angular pressing as a processing tool for grain refinement. Prog. Mater. Sci..

[14] Tang Y., Sumikawa K., Takizawa Y., Yumoto M., Otagiri Y., Horita Z. (2019). Multi-pass high-pressure sliding (MP-HPS) for grain refinement and superplasticity in metallic round rods. Mater. Sci. Eng. A.

[15] Tóth L.S., Arzaghi M., Fundenberger J.J., Beausir B., Bouaziz O., Arruffat-Massion R. (2009). Severe plastic deformation of metals by high-pressure tube twisting. Scr. Mater..

[16] Kunčická L., Král P., Dvořák J., Kocich R. (2019). Texture evolution in biocompatible mg-y-re alloy after friction stir processing. Metals.

[17] Zisman A.A., Rybin V.V., Van Boxel S., Seefeldt M., Verlinden B. (2006). Equal channel angular drawing of aluminium sheet. Mater. Sci. Eng. A.

[18] Kocich R., Macháčková A., Andreyachshenko V.A. (2015). A study of plastic deformation behaviour of Ti alloy during equal channel angular pressing with partial back pressure. Comput. Mater. Sci..

[19] Fatemi-Varzaneh S.M., Zarei-Hanzaki A., Izadi S. (2010). Shear deformation and grain refinement during accumulative back extrusion of AZ31 magnesium alloy. J. Mater. Sci..

[20] Kocich R., Macháčková A., Fojtík F. (2012). Comparison of strain and stress conditions in conventional and ARB rolling processes. Int. J. Mech. Sci..

[21] Mohebbi M.S., Akbarzadeh A. (2010). Accumulative spin-bonding (ASB) as a novel SPD process for fabrication of nanostructured tubes. Mater. Sci. Eng. A.

[22] Gupta A.K., Maddukuri T.S., Singh S.K. (2016). Constrained groove pressing for sheet metal processing. Prog. Mater. Sci..

[23] Torkestani A., Dashtbayazi M.R. (2018). A new method for severe plastic deformation of the copper sheets. Mater. Sci. Eng. A.

[24] Abdolvand H., Sohrabi H., Faraji G., Yusof F. (2015). A novel combined severe plastic deformation method for producing thin-walled ultrafine grained cylindrical tubes. Mater. Lett..

[25] Zangiabadi A., Kazeminezhad M. (2011). Development of a novel severe plastic deformation method for tubular materials: Tube Channel Pressing (TCP). Mater. Sci. Eng. A.

[26] Wang C., Li F., Li Q., Li J., Wang L., Dong J. (2013). A novel severe plastic deformation method for fabricating ultrafine grained pure copper. Mater. Des..

[27] Wang Q.D., Chen Y.J., Lin J.B., Zhang L.J., Zhai C.Q. (2007). Microstructure and properties of magnesium alloy processed by a new severe plastic deformation method. Mater. Lett..

[28] Kim K., Yoon J. (2013). Evolution of the microstructure and mechanical properties of AZ61 alloy processed by half channel angular extrusion (HCAE), a novel severe plastic deformation process. Mater. Sci. Eng. A.

[29] Richert M., Liu Q., Hansen N. (1999). Microstructural evolution over a large strain range in aluminium deformed by cyclic-extrusion–compression. Mater. Sci. Eng. A.

[30] Ghosh A.K., Huang W. (2000). Severe Deformation Based Process for Grain Subdivision and Resulting Microstructures. Investigations and Applications of Severe Plastic Deformation.

[31] Pardis N., Talebanpour B., Ebrahimi R., Zomorodian S. (2011). Cyclic expansion-extrusion (CEE): A modified counterpart of cyclic extrusion-compression (CEC). Mater. Sci. Eng. A.

[32] Ensafi M., Faraji G., Abdolvand H. (2017). Cyclic extrusion compression angular pressing (CECAP) as a novel severe plastic deformation method for producing bulk ultrafine grained metals. Mater. Lett..

[33] Khoddam S., Farhoumand A., Hodgson P.D. (2011). Axi-symmetric forward spiral extrusion, a kinematic and experimental study. Mater. Sci. Eng. A.

[34] Farhoumand A., Hodgson P.D., Khoddam S. (2013). Finite element analysis of plastic deformation in variable lead axisymmetric forward spiral extrusion. J. Mater. Sci..

[35] Ebrahimi G.R., Barghamadi A., Ezatpour H.R., Amiri A. (2019). A novel single pass severe plastic deformation method using combination of planar twist extrusion and conventional extrusion. J. Manuf. Process..

[36] Yu J., Zhang Z., Wang Q., Hao H., Cui J., Li L. (2018). Rotary extrusion as a novel severe plastic deformation method for cylindrical tubes. Mater. Lett..

[37] Mizunuma S. (2006). Large Straining Behavior and Microstructure Refinement of Several Metals by Torsion Extrusion Process. Mater. Sci. Forum.

[38] Noor S.V., Eivani A.R., Jafarian H.R., Mirzaei M. (2016). Inhomogeneity in microstructure and mechanical properties during twist extrusion. Mater. Sci. Eng. A.

[39] Pardis N., Ebrahimi R. (2009). Deformation behavior in Simple Shear Extrusion (SSE) as a new severe plastic deformation technique. Mater. Sci. Eng. A.

[40] Utsunomiya H., Hatsuda K., Sakai T., Saito Y. (2004). Continuous grain refinement of aluminum strip by conshearing. Mater. Sci. Eng. A.

[41] Lee J.-C., Seok H.-K., Suh J.-Y. (2002). Microstructural evolutions of the Al strip prepared by cold rolling and continuous equal channel angular pressing. Acta Mater..

[42] Huang Y., Prangnell P.B. (2007). Continuous frictional angular extrusion and its application in the production of ultrafine-grained sheet metals. Scr. Mater..

[43] Huang J.Y., Zhu Y.T., Jiang H., Lowe T.C. (2001). Microstructures and dislocation configurations in nanostructured Cu processed by repetitive corrugation and straightening. Acta Mater..

[44] Nakamura K., Neishi K., Kaneko K., Nakagaki M., Horita Z. (2004). Development of Severe Torsion Straining Process for Rapid Continuous Grain Refinement. Mater. Trans..

[45] Kocich R., Lukáč P. (2015). SPD Processes-Methods for Mechanical Nanostructuring. Handbook of Mechanical Nanostructuring.

[46] Semenova I.P., Polyakov A.V., Raab G.I., Lowe T.C., Valiev R.Z. (2012). Enhanced fatigue properties of ultrafine-grained Ti rods processed by ECAP-Conform. J. Mater. Sci..

[47] Kocich R., Greger M., Kursa M., Szurman I., Macháčková A. (2010). Twist channel angular pressing (TCAP) as a method for increasing the efficiency of SPD. Mater. Sci. Eng. A.

[48] Jamili A.M., Zarei-Hanzaki A., Abedi H.R., Mosayebi M., Kocich R., Kunčická L. (2019). Development of fresh and fully recrystallized microstructures through friction stir processing of a rare earth bearing magnesium alloy. Mater. Sci. Eng. A.

[49] Kocich R., Fiala J., Szurman I., Macháčková A., Mihola M. (2011). Twist-channel angular pressing: Effect of the strain path on grain refinement and mechanical properties of copper. J. Mater. Sci..

[50] Iqbal U.M., Muralidharan S. (2019). Optimization of die design parameters and experimental validation on twist channel angular pressing process of AA6061-T6 aluminium alloy. Mater. Res. Express.

[51] Segal V.M. (2003). Slip line solutions, deformation mode and loading history during equal channel angular extrusion. Mater. Sci. Eng. A.

[52] Kim H.S., Seo M.H., Hong S.I. (2000). On the die corner gap formation in equal channel angular pressing. Mater. Sci. Eng. A.

[53] Orlov D., Beygelzimer Y., Synkov S., Varyukhin V., Horita Z. (2008). Evolution of Microstructure and Hardness in Pure Al by Twist Extrusion. Mater. Trans..

[54] Latypov M.I., Lee M.G., Beygelzimer Y., Kulagin R., Kim H.S. (2015). On the simple shear model of twist extrusion and its deviations. Met. Mater. Int..

[55] Bahadori S.R., Dehghani K., Akbari Mousavi S.A.A. (2015). Comparison of microstructure and mechanical properties of pure copper processed by twist extrusion and equal channel angular pressing. Mater. Lett..

[56] Kocich R., Kunčická L., Mihola M., Skotnicová K. (2013). Numerical and experimental analysis of twist channel angular pressing (TCAP) as a SPD process. Mater. Sci. Eng. A.

[57] Glezer A.M., Sundeev R.V. (2015). General view of severe plastic deformation in solid state. Mater. Lett..

[58] Kunčická L., Kocich R., Král P., Pohludka M., Marek M. (2016). Effect of strain path on severely deformed aluminium. Mater. Lett..

[59] Kocich R., Kunčická L., Král P., Macháčková A. (2016). Sub-structure and mechanical properties of twist channel angular pressed aluminium. Mater. Charact..

[60] Stolyarov V.V., Zhu Y.T., Alexandrov I.V., Lowe T.C., Valiev R.Z. (2001). Influence of ECAP routes on the microstructure and properties of pure Ti. Mater. Sci. Eng. A.

[61] Kunčická L., Kocich R. (2017). Structure Development after Twist Channel Angular Pressing. Acta Phys. Pol. A.

[62] Kunčická L., Kocich R., Ryukhtin V., Cullen J.C.T., Lavery N.P. (2019). Study of structure of naturally aged aluminium after twist channel angular pressing. Mater. Charact..

[63] Beyerlein I.J., Tóth L.S. (2009). Texture evolution in equal-channel angular extrusion. Prog. Mater. Sci..

[64] Kocich R., Macháčková A., Kunčická L. (2014). Twist channel multi-angular pressing (TCMAP) as a new SPD process: Numerical and experimental study. Mater. Sci. Eng. A.

[65] Kocich R., Kunčická L., Macháčková A. (2014). Twist Channel Multi-Angular Pressing ( TCMAP ) as a method for increasing the efficiency of SPD. IOP Conf. Ser. Mater. Sci. Eng..

[66] Alavizadeh S.M., Abrinia K., Parvizi A. (2020). Twisted Multi Channel Angular Pressing (TMCAP) as a Novel Severe Plastic Deformation Method. Met. Mater. Int..

[67] Shamsborhan M., Ebrahimi M. (2016). Production of nanostructure copper by planar twist channel angular extrusion process. J. Alloys Compd..

[68] Shokuhfar A., Shamsborhan M. (2014). Finite element analysis of planar twist channel angular extrusion (PTCAE) as a novel severe plastic deformation method. J. Mech. Sci. Technol..

[69] Shamsborhan M., Shokuhfar A., Nejadseyfi O., Kakemam J., Moradi M. (2015). Experimental and numerical comparison of equal channel angular extrusion (ECAE) with planar twist channel angular extrusion (PTCAE). Proc. Inst. Mech. Eng. Part C.

